# Inflammation and Fibrogenesis in MAFLD: Role of the Hepatic Immune System

**DOI:** 10.3389/fmed.2021.781567

**Published:** 2021-12-09

**Authors:** Pietro Torre, Benedetta Maria Motta, Roberta Sciorio, Mario Masarone, Marcello Persico

**Affiliations:** ^1^Internal Medicine and Hepatology Unit, Department of Medicine, Surgery and Dentistry, “Scuola Medica Salernitana”, University of Salerno, Salerno, Italy; ^2^Department of Medicine, Surgery and Dentistry, “Scuola Medica Salernitana”, University of Salerno, Baronissi, Italy

**Keywords:** NAFLD, MAFLD, liver immunology, immunometabolism, liver fibrogenesis, NAFLD therapies

## Abstract

Metabolic (dysfunction)-associated fatty liver disease (MAFLD) is the definition recently proposed to better circumscribe the spectrum of conditions long known as non-alcoholic fatty liver disease (NAFLD) that range from simple steatosis without inflammation to more advanced liver diseases. The progression of MAFLD, as well as other chronic liver diseases, toward cirrhosis, is driven by hepatic inflammation and fibrogenesis. The latter, result of a “*chronic wound healing reaction*,” is a dynamic process, and the understanding of its underlying pathophysiological events has increased in recent years. Fibrosis progresses in a microenvironment where it takes part an interplay between fibrogenic cells and many other elements, including some cells of the immune system with an underexplored or still unclear role in liver diseases. Some therapeutic approaches, also acting on the immune system, have been probed over time to evaluate their ability to improve inflammation and fibrosis in NAFLD, but to date no drug has been approved to treat this condition. In this review, we will focus on the contribution of the liver immune system in the progression of NAFLD, and on therapies under study that aim to counter the immune substrate of the disease.

## Introduction: Definitions, Change of Terminology, and Epidemiology

MALFD stands for “*metabolic (dysfunction)-associated fatty liver disease*” and is a recently recommended term by an international panel of experts (1) to replace the long used NAFLD (non-alcoholic fatty liver disease) and NASH (non-alcoholic steatohepatitis). The latter was coined by Ludwig et al., referring to the fatty liver and inflammation observed in biopsy specimens of patients who had other metabolic disorders, such as obesity or related conditions, and were not alcohol abusers (2), while NAFLD appeared for the first time in a paper by Schaffner and Thaler (3). According to EASL and AASLD guidelines, NAFLD indicates an excessive accumulation of liver fat, corresponding to the presence of steatosis in >5% of hepatocytes, documented by liver histology or imaging, in people who don't drink an at-risk amount of alcohol (nor having other causes of steatosis). The latter specification is believed to represent a weak point in this definition, due to the absence of an international consensus in defining threshold levels for at-risk alcohol consumption and the potential shame associated with the term “alcoholic” (1, 4–6). NAFLD term includes a set of pathological conditions ranging from mild alterations (NAFL) to others conferring a worse prognosis (NASH, which implies hepatocyte injury and liver fibrosis of increasing severity up to NASH-cirrhosis, and hepatocarcinoma). On the other hand, the Asian Pacific Association for the Study of the Liver (APASL) has already presented guidelines on the diagnosis and management of MAFLD (7). The open challenge is to find out the causes why some people with NAFLD progress to advanced liver disease while others do not (8).

The agreement on the term “MAFLD” originated to emphasize the metabolic etiology of this spectrum of conditions, and consequently avoid the use of a “non-definition” (1). Moreover, with this new term the coexistence of other cofactors for the progression of the disease, including alcohol consumption, is allowed (1, 6). Experts proposed that the term MAFLD should include the set of conditions, overcoming the non-NASH/NASH dichotomy and that it should be enriched with data on the severity of the disease (grade of activity and stage of fibrosis) (1). The issue of the terminology of NAFLD and NASH is not new, in fact was already addressed in the past (1, 9). However, skepticism is not absent regarding the recently proposed change, which according to some authors could be precocious and counterproductive (10). Their doubts concern the use of a term (“metabolic”) that likewise may lack specificity; because other liver diseases (also responsible for hepatic steatosis, e.g., Wilson disease) have a metabolic etiology; since, although knowledge of pathophysiology and other aspects of NAFLD has increased, great challenges still exist; furthermore, they believe this change could have negative repercussions for socio-sanitary and scientific reasons (10).

NAFLD is estimated to have a global prevalence of around 25% of the general population and is responsible for high morbidity and mortality, having been found that its prevalence has grown in tandem with the global increase of obesity (8, 11–13). It is an increasingly common cause of liver transplantation and hepatocarcinoma, which in NAFLD can arise even in the absence of cirrhosis (8). In addition to liver-related causes of morbidity and mortality, it has a strong link with the various components of the metabolic syndrome (MetS) (8); in fact, NAFLD showed to have a high prevalence in patients with MetS elements (12, 14). It was also observed that, over the years, people with NAFLD have a high probability of developing other metabolic comorbidities, cardiovascular diseases, and non-fatal or fatal events (the latter representing the leading cause of death for these patients), compared to those without NAFLD (8, 13, 15, 16), and that patients with cardiovascular disease (CVD) risk factors have an increased risk of developing NAFLD compared to people without these risk factors, suggesting a bi-directional relationship between NAFLD and CVD risk factors (15). The cardiovascular risk for patients with NAFLD, which is especially observed for those who have NASH, appears to be independent of the various components of the metabolic syndrome, suggesting a direct role of the liver disease (16, 17). Despite its strong negative impact on human health, to date, there are still no approved therapies to reverse this condition. The chronic inflammation which occurs in NASH is a central pathophysiological event and guides the progression of the disease through increasing degrees of fibrosis toward liver cirrhosis.

## Homeostasis of the Liver Immune System in Health State

The liver is crucial in the metabolism of carbohydrates, lipids, and proteins, is responsible for bile formation, detoxification and inactivation of substances, and has storage functions, but it is also an important immune organ (18). In the hepatic parenchyma, a rich variety of elements participating in the immune response exists (19), some of which being not strictly immune cells. Among the latter there are hepatocytes, the most abundant cell population of the liver, which, in addition to their “primary” functions, express Pattern Recognition Receptors (PRRs), can produce acute phase proteins, cytokines, chemokines, complement proteins and other opsonins; they produce proteins involved in iron metabolism, such as hepcidin, the availability of this element being able to affects bacterial proliferation; hepatocytes are the main source of LPS-binding protein, soluble CD14, and soluble MD-2, which participate in the formation of TLR4-MD-2-LPS complex, from which, in turn, starts the signaling that leads to NF-kB activation and inflammatory responses; fibrinogen, produced by hepatocytes, participates in the immune response as it mediates the adhesion of leukocytes, can activate the complement system, and because its active fragment fibrin has antibacterial properties; moreover, they express MHC-I and in some conditions also MHC-II, lacking, however, in the expression of costimulatory molecules (18–22), liver sinusoidal endothelial cells (LSECs, which in addition to offering a physical barrier between the lumen of the sinusoid and the space of Disse, participate in the process of leukocytes transmigration, exhibit scavenger activity, are capable of endocytosis, express TLRs and MHC molecules, and are involved in tolerance mechanisms, by direct action on T lymphocytes, e.g., by PDL1 expression, or through the “veto” function, consisting in vetoing the ability of other APCs, like dendritic cells, to activate T lymphocytes, in a mode requiring physical contact but MHC-independent) (19, 23–28), biliary epithelial cells (BECs, antigen presentation, TLRs expression, production of inflammatory mediators in response to insults; these cells were found capable of “endotoxin tolerance,” which was demonstrated after observation that human intrahepatic biliary epithelial cell lines pretreated with LPS developed tolerance to further stimulation with such substance; this effect was attributed to the negative regulation of the TLR signaling mediated by interleukin-1 receptor-associated kinase M, IRAK-M) (19, 29), and hepatic stellate cells (HSCs, the main actors in the fibrogenesis process, also express TLRs, MHC-I, MHC-II, and CD1 molecules, and, as observed for the LSECs, are involved in the induction of T-cell tolerance also through a veto function) (19, 22, 30). These cells are part of innate immunity, but also interact with elements of adaptive responses. Among the innate immune cells housed in liver sinusoids there are myeloid- (Kupffer cells, KC, dendritic cells, DCs, myeloid-derived suppressor cells, MDSC) or lymphoid-derived cells (such as natural killers, NK, and innate lymphoid cells, ILCs). Other abundant elements do not reflect either the innate or adaptive system criteria and were therefore defined as “innate-like,” or “unconventional” lymphocytes. These include mucosal-associated invariant T (MAIT) cells, which are today deemed to be a leading share of hepatic T lymphocytes in the healthy liver (31), natural killer T (NKT) cells, and γδ-T cells. Furthermore, the healthy liver also hosts conventional T and B lymphocytes (adaptive immunity) (19). Compared to other lymphoid organs, such as the lymph nodes and the spleen, the liver greatly differs in terms of composition of its resident cells (32).

A complex relationship between the large number of antigens to which cells of healthy liver are continuously exposed and the maintenance of an immune homeostasis exists: the liver occupies a first-line position, filtering more than 2,000 liters of blood per day coming from the portal vein, which in turns carries a large amount of gut-derived food antigens and bacterial products (e.g., LPS), and from the hepatic artery which transports oxygen-rich blood (22). Furthermore, in the liver it occurs the formation of neo-antigens due to the intrahepatic transformation of many compounds (32). Under stationary conditions, the hepatic immune cells maintain tolerance to non-harmful substances (e.g., food-derived antigens), but they must also be able to mount an adequate response against the pathogenetic ones (22, 32). The tolerance state originates in a tolerogenic microenvironment, due to the complex interplay that takes place between different cells. In fact, liver resident cells block adaptive immune responses by inducing states of energy, exhaustion, deviation, or by leading immune cells to apoptosis (22, 33). The concept of hepatic tolerance was initially hypothesized in the 60's by observing long-term survivals of allogeneic pig liver transplants without using immunosuppression (34, 35), a phenomenon subsequently confirmed in other animal models (36). Furthermore, the finding that liver transplanted animals receiving non-hepatic allografts from the same liver donor showed acceptance of such grafts, suggested that the liver can induce systemic T-cell tolerance (36).

Among the mechanisms responsible for liver immune tolerance, there is the expression by liver cells of MHC complexes in the absence of costimulatory molecules (e.g., CD80/CD86); lack of MHC-II expression; release of cytokines with suppressor activity, such as IL 10 or TGF-β; exposure of immune cells to programmed cell death ligand-1 (PD-L1), or Fas-L; phagocytosis by Kupffer cells; inhibition of professional APC activating function (19, 30, 32, 37, 38). In the context of liver transplants specific mechanisms inducing tolerance take part (36). Since tolerance has been observed to be a marked phenomenon in the liver, hypotheses have been formulated to explain this occurrence (33). In the “*graveyard hypothesis*” the liver was conceived as a site where T lymphocytes that are already directed toward apoptosis (“moribund” lymphocytes) are sequestered, whereas the “*killing field hypothesis*” suggests that this organ may be a site in which activated T lymphocytes accumulate, and where tolerance mechanisms lead them to apoptosis (39). The “*school*” model was another suggested theory, and postulates that lymphocytes migrating through the liver are educated (like “students”) to have regulatory functions rather than participate in immunosurveillance; in this model, the hepatic antigen presenting cells (hepatocytes, LSECs, KCs, DCs, HSCs) represent the “teachers” who induce such lymphocytes to a regulatory state, this action being favored by the anatomy of the hepatic sinusoids (40). Another modality of hepatic immune homeostasis maintenance was observed to depend on liver draining lymph nodes (LNs), differently depending on which one is considered, having been found that portal LN is a site of regulatory T cells induction, whereas the celiac LN is involved in T cell responses (33, 41).

Bile acids and the extracellular matrix (as described below) can also modulate the immune response in the liver (19, 42, 43). Moreover, cellular metabolism is closely linked to immune properties. In fact, different metabolic patterns have been found associated with different immune cell functions. A predominantly glycolytic metabolism was observed in different types of effector T lymphocytes and other activated immune cells with effector function participating in inflammatory processes, while fatty acid oxidation was observed to be preferred by non-inflammatory immune cells (e.g., regulatory T cells, T_reg_) (44, 45). Moreover, it was observed that glycolysis induced by HIF-1α on the one hand, and oxidative metabolism induced by IL-4 / STAT6 / PGC-1β on the other hand, drove different types of macrophage phenotypes, proinflammatory (46) vs. alternative (anti-inflammatory) (47), respectively (48).

In contrast to the immune homeostasis of the healthy liver, which nevertheless is capable of effective local or systemic inflammatory responses, in NAFLD, cells with immune functions become key players in the disease progression.

## NAFLD is a Multifactorial, Systemic Disease Caused by a Set of Simultaneous and Synergistic Events

The “two-hit” model for NAFLD progression was proposed in 1998 by C. P. Day and O. F. James. In this theory, the first hit is the excess in the accumulation of lipids within hepatocyte (steatosis), and the second one corresponds to other factors responsible for steatohepatitis (49). The currently accepted theory, “multiple-hit hypothesis,” proposed by Tilg and Moschen (50), replaced the two-hit model and indicates that there are multiple synergistic events leading to liver inflammation, proceeding in parallel. In this theory, inflammation not necessarily follows the fat accumulation, being the opposite also plausible: inflammation caused by different insults could exist before steatosis in NASH, and may contribute to its progression (50). Several factors contribute to this pathological condition, including insulin resistance, which is a central event in the NAFLD pathophysiology, excess flow of fatty acids to the liver, lipotoxicity, mitochondrial dysfunction, oxidative stress, endoplasmic reticulum stress (50, 51). Altered liver-adipose tissue cross talk (because of the effect on the liver of the imbalance of adipokine production by a dysfunctional adipose tissue) (50, 51) and gut-liver axis, are important dysfunctions occurring in NAFLD and implicated in its pathogenesis (50). Patients with NAFLD showed to have changes in gut microbiota, a high prevalence of intestinal bacterial overgrowth, and increased gut permeability (52–54). The increased liver exposure to bacterial derived products (e.g., endotoxemia), proved to cause liver fat accumulation and inflammation mediated by immune system cells (e.g., Kupffer cells *via* TLR-4) (51, 55). Among the genetic factors conferring susceptibility to NAFLD there are polymorphisms in *patatin-like phospholipase domain containing-3* (*PNPLA3*) gene, which is the most studied in NAFLD, *transmembrane 6 superfamily, member 2* (*TM6SF2*) gene, *membrane bound O-acyltransferase domain containing 7-transmembrane channel-like 4* (*MBOAT7*) gene, *glucokinase regulator (GCKR)* gene, and *17-beta hydroxysteroid dehydrogenase-13 (HSD17B13)* gene (8, 56); moreover, variants of genes regulating the mitochondrial activity, insulin signaling, and immune response have also been shown to be involved in such disease (57). Epigenetic changes, such as altered DNA methylation and miRNA expression, have recently been investigated in NAFLD and linked to disease progression (58–60). Environmental risk factors affect the onset and progression of fatty liver and include dietary styles like Western diet (high in saturated fats), high consumption of fructose (e.g., that contained in some sweetened beverages or high fructose corn syrup) and refined carbohydrates, and sedentary lifestyle. The prevalence of NAFLD also varies in relation to age, sex, and ethnicity (4, 8, 51, 61). It should be noted, however, that not a single risk factor but the interplay of many elements causes NAFLD progression; in fact, not all obese or people with risk factors for NAFLD are affected by this condition, and NAFLD can develop in non-obese, non-diabetic people (8).

The concept of metabolic flexibility (opposed to metabolic inflexibility) indicates the ability to adjust the utilization of substrates depending on different conditions (e.g., changes in their availability) (8, 62). The typical alterations observed in NAFLD patients (high triglycerides, FFAs, and insulin) led to the hypothesis that it could be a condition characterized by metabolic inflexibility (8). A key element for the pathogenesis of NAFLD is the excess of fat and lipotoxicity (51, 63, 64). The latter, rather than the excess of fat alone, is associated with disease progression (65). The excess of circulating FFAs and the consequent abnormal liver uptake and fat accumulation typical of NAFLD, derives from abnormal lipolysis (hydrolysis of triglyceride) in the adipose tissue, mediated by insulin resistance, which is the event responsible for the largest share of hepatic fat accumulation, increased *de novo* lipogenesis (starting from glucose or fructose), and excess in dietary fat intake (64, 66, 67). Fatty acids in the liver are addressed to oxidation (mitochondrial β-oxidation, or oxidation in peroxisomes, or microsomes) or are esterified to triglycerides (TGs), to form very low-density lipoprotein (VLDL) particles, which will be secreted, or lipid droplets, which will be stored in the hepatocytes (63, 64, 67). Triglycerides formation, although associated with steatosis, is thought to be a protective response to an excess of fats, as it will be stored in an inert, non-toxic form (50, 63, 67, 68). Saturation of the processes responsible FFAs handling, due to the large amount that reaches the hepatic parenchyma, leads to alterations in mitochondrial function and an increase in the production of reactive oxygen species (ROS) (69). This ROS increase is not effectively counteracted and in NAFLD it was found an inefficiency of the ROS detoxification systems (70). The resulting oxidative stress also causes lipid damage by lipid peroxidation, which results in the formation of compounds (e.g., 4-hydroxy-2-nonenal, 4-HNE, and malondialdehyde, MDA) that contribute to the disease progression (69, 71, 72). However, it is still unclear whether mitochondrial dysfunction is a consequence of NAFLD-associated alterations or an upstream condition that predisposes to NASH (66, 73). The oxidative stress that occurs in NAFLD is in close association with activation of the immune system, e.g., ROS are a stimulus for the activation of Kupffer cells (KCs), which in turn will become ROS producers (74, 75). Lipotoxicity refers to cell dysfunctions and injury caused by lipids; saturated fatty acids such as palmitic acid and stearic acid, lysophosphatidylcholine, free cholesterol, and ceramides are considered lipotoxic species (65, 76, 77). Lipotoxicity leads to endoplasmic reticulum stress, altered autophagy, release of extracellular vehicles (EVs), and, ultimately, to activation of cell death pathway (64, 67, 78, 79). EVs, which are distinguished by size in exosomes (up to 100 nm in diameter) and microparticles (from 100 to 1,000 nm), are involved in cell-cell communication (80), and during lipotoxicity-induced hepatocytes injury they would contribute to the liver damage by eliciting pro-inflammatory responses [e.g., by inducing the release of inflammatory cytokines in macrophages (65, 81–83); moreover, they were found to be internalized by HSCs and cause their activation (81)]. Given their role in NAFLD, EVs were proposed as a marker of diseases progression (65).

**Figure 1:**
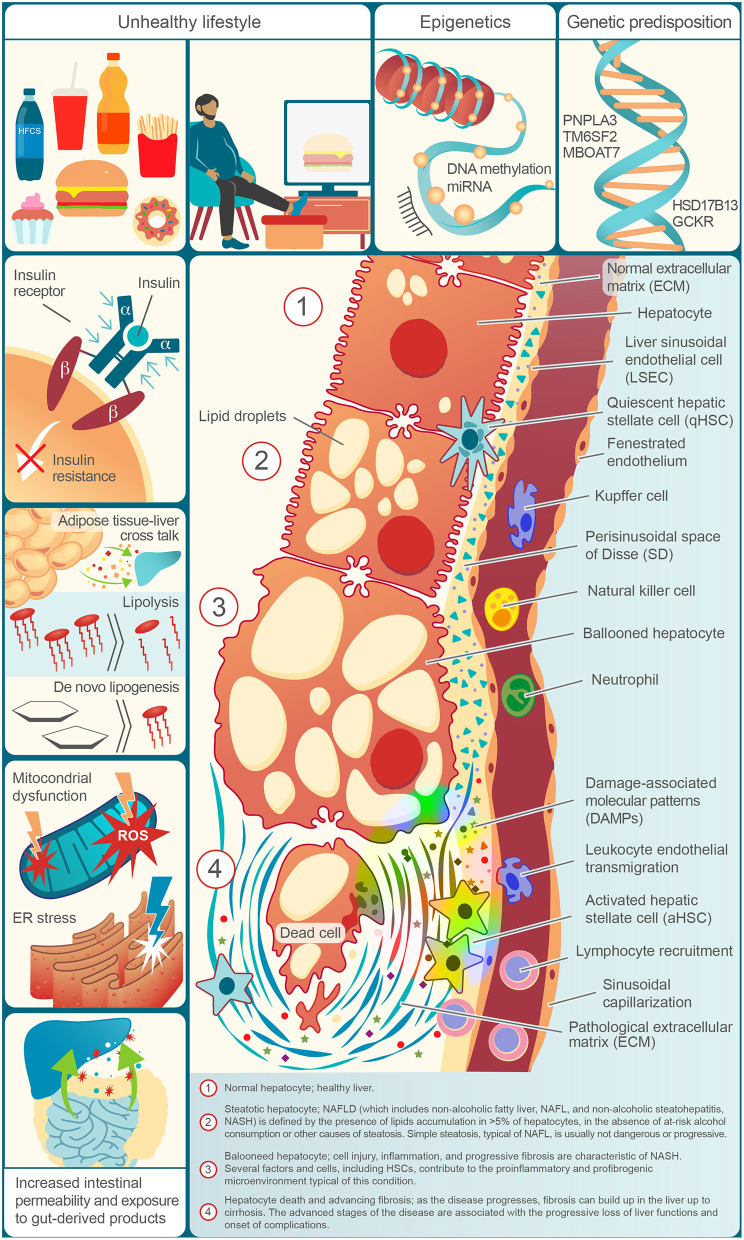
Risk factors, physiopathological molecular events, and typical elements of the NAFLD spectrum in a decorative, cell by cell, succession.

Figure 1 illustrates the risk factors for NAFLD, the molecular events underlying its progression, and the histological features found in the distinct entities of its spectrum.

## The Fibrogenesis Process in NAFLD

The hepatocytes injury and death, caused in NAFLD by metabolic dysfunctions, lead to the release of warning signals which are responsible for recruitment and activation of immune and fibrogenic cells. These cells amplify the pathological process by releasing pro-inflammatory and pro-fibrogenic factors, thus creating a vicious circle (69, 84–88).

Fibrogenesis has the physiological role of repairing a damaged tissue, so acting as a wound healing response. However, regardless of etiology, chronic liver injury and inflammation and the consequent fibrogenesis, over the years, can lead to progressive fibrosis, which in turn can evolve to liver cirrhosis, a silent condition until its complications appear, which is associated with high morbidity and mortality (8, 65). Abnormal hepatic fibrogenesis is a dynamic process in which an excess of production and a progressive accumulation over time of extracellular matrix (ECM) components takes part. In fact, in pathological conditions, the regulation of the amount of matrix, as a result of deposition and reabsorption processes, is not guaranteed (65).

Normal ECM is composed of different classes of components, including several types of fibrillary and non-fibrillar collagens, non-collagenous proteins (such as fibronectin, laminin, and elastin), and proteoglycans (89). In a proteomics study of healthy liver tissue samples, it was observed that the ECM is made up of more than 100 distinct ECM proteins (90). In physiological conditions, ECM is directly produced by many cell types (HSCs, hepatocytes, LSECs, cholangiocytes) (91). Furthermore, these cells release matrix metalloproteinase (MMPs), the major class of enzymes responsible for ECM degradation, and tissue inhibitors of metalloproteinases (TIMPs). For the maintenance of homeostasis, there is a fine balance between the activity of MMPs and that of TIMPs (92). In an experimental model of liver fibrosis, increased activity of TIMP-1 was found to be associated with a decreased spontaneous hepatic fibrosis resolution (93). In the healthy liver, anyway, the ECM occupies only a small part of the entire parenchyma; in the space of Disse, it forms a thin and discontinuous layer (94).

HSCs are the main source of ECM-producing fibroblasts (65, 89). In normal liver, these cells are localized in the space of Disse, and by their dendritic processes, they interact with hepatocytes and other adjacent elements of the liver parenchyma (65). Here they are involved in ECM homeostasis, work as a deposit of vitamin A (of which they are the main repository), and have immune functions (65, 95). After activation and trans-differentiation, they transform into myofibroblast-like cells (HSC/MFs) which abundantly proliferate and produce EC matrix, migrate in response to chemoattractants, produce proinflammatory mediators, thus directly contributing to the “profibrogenic environment,” and have more marked contractile properties (65). Their contraction can also influence the portal pressure (96). In addition to HSCs, a smaller proportion of fibrogenic cells derives from portal fibroblasts, but other cells of origin have also been described, such as bone marrow-derived precursor, hepatocytes and cholangiocytes (reflecting a process of “epithelial-to-mesenchymal transition,” EMT) (97), or mesothelial cells (MCs), although the contribution of these cells to liver MFs is believed to be minor or questionable (65). It has been hypothesized that ECM and the activity of the HSCs during NASH could have a beneficial role in the early stages of disease being, on the other hand, detrimental in later stages (89).

Activation of HSCs includes initiation and progression phases, occurs in an inflammatory context, and depends on the interaction with many elements, including immune system cells which promote and sustain the fibrogenesis process by producing several mediators (65, 91). Among these, a crucial role is played by profibrogenic cytokines. Transforming growth factor-β (TGFβ) is released by different cell types and is considered the most potent fibrogenic cytokine and activator of HSCs, leading to the production of type I collagen through a signaling pathway that involves Smad proteins (65, 89, 98–100). Phagocytosis of apoptotic cells by macrophages was found *in vitro* to increase the release of TGFβ (101), and also the HSCs were found capable of phagocytizing apoptotic bodies, this event having been found to be causative of profibrogenic responses (102); these results defined a link between hepatocytes death and fibrogenesis (102). Platelet-derived growth factor (PDGF) is another pro-fibrogenic cytokine, which leads to the proliferation and migration of HSCs (100, 103). Other cytokines involved in HSCs activation or proliferation include VEGF, CTGF, and IL-17 (65, 89, 100, 104). Furthermore, leptin showed to exert profibrotic effects, while adiponectin exhibited antifibrogenic properties (105).

Damaged or dead hepatocytes during NAFLD release damage-associated molecular patterns (DAMPs), which can activate the HSCs by toll-like receptors (TLRs) (89, 106), and TLRs on HSCs also perceive microbial products (such as LPS), which increase due to the altered intestinal permeability associated with NAFLD, resulting in activation of these cells (100, 107, 108). Hepatocyte derived hedgehog (Hh) ligands and osteopontin (OPN) were found capable of activating HSCs in NAFLD (109, 110). Another emerging signaling pathway for HSCs activation is the Hippo pathway, which involves the Yes-associated protein (YAP) (89, 111). Among the different stimuli that have been found to activate the HSCs (65, 112), there is the accumulation in these cells of free cholesterol (FC), which was found to lead to an increase of TLR4 expression and to sensitize HSCs to the action of TGFβ (113).

ECM is considered an active biological system, with immunomodulating properties. In fact, it can directly influence the activity of cells participating in the progression of NAFLD. Some components of the ECM include domains that can interact with immune system receptors, having anti- or pro-inflammatory effects. For example, collagen is recognized by the leukocyte associated immune receptor (LAIR)−1, which is expressed by most immune cells and induce a state of immunosuppression, but depending on its expression level and interaction with other molecules (e.g., soluble LAIR-2), it may also lead to pathological states (91, 114). ECM components were also found to directly influence the activity of HSCs through integrins and discoidin domain-containing receptors (DDRs) (100, 115). Moreover, ECM fragments produced during tissue damage, or components actively secreted, can act as DAMPs being recognized by immune system cells through PRRs (91, 116); DAMP-ECM derived responses were found to be mediated primarily by TLR2 and TLR4 (116). ECM components that have been associated with pathological responses include versican (whose mRNA was found to be upregulated in rats with NAFLD, and in biopsies of patients with advanced fibrosis; circulating versican levels were found increased in serum of patients with advanced fibrosis) (117, 118), thrombospondin-1 (TSP-1; in an *in vitro* NAFLD model intracellular lipid accumulation was found associated to TSP mRNA upregulation) (119), cysteine-rich protein 61 (CCN1, which induced hepatic inflammation and injury in a mouse model of NAFLD) (120), lumican (whose hepatic expression was found to be high in patients with progressive NAFLD) (121), and periostin (whose circulating and tissue levels were found to be higher in NAFLD patients than controls) (122, 123). These ECM components can induce the release of pro-inflammatory cytokines, recruitment and activation of immune cells (91). Other components, on the other hand, have shown anti-inflammatory and anti-fibrotic properties (e.g., Extracellular Matrix Protein-1, ECM1, and High Molecular Weight-Hyaluronic Acid, HMW-HA) (91). Studies have shown a link between their genetic depletion and liver fibrosis progression or their immunosuppressive properties, e.g., through the support of the function of regulatory T lymphocytes (91, 124, 125). Moreover, other ECM constituents may have both a pro- or an anti-inflammatory role based on temporal (i.e., stage of the disease) and spatial factors, and depending on the type of receptor or cell from which they are recognized (91). In addition to these effects, ECM is a storage site for cytokines and growth factors (126).

In case of fibrosis and cirrhosis beyond the quantity, also the composition of the ECM is altered (65). In fact, in a healthy liver, the ECM that surrounds the hepatocytes was found to be formed mainly by type IV collagen, laminins, and proteoglycans, while in a liver with fibrosis fibrillar collagen types I and III become prevalent (65, 127). Although in the fibrogenesis process several factors are etiology-independent (127), the progression of fibrosis in chronic liver disease proceeds differently based on the cause. NAFLD, as well as alcoholic steatosis progressing to steatohepatitis, typically has a perisinusoidal (matrix deposition around the sinusoid) and pericellular (around groups of hepatocytes) pattern of fibrogenesis (65, 127).

## The Immune System in the Progression of NAFLD

The immune system plays a key role in hepatic fibrogenesis, as it supports the inflammation that precedes and accompanies the fibrogenic process (128). Recent works have highlighted the link between metabolic dysregulation and activation of the immune system (129–131). As mentioned above, different functions of the immune system are associated with different cellular metabolic activities (e.g., glycolytic vs. oxidative). As hypothesized in a recent review by Cai et al., the altered systemic metabolism that is found in metabolic diseases, characterized by changes in the availability of substrates or presence of specific compounds, could affect the activity of the immune cells by changes in cellular metabolism (132). The recently described *trained immunity* (TI) or *innate immune memory* (long lasting, although less than the adaptive immune system memory, increased responsiveness of cells of the innate system, e.g., monocytes, following secondary stimulations with an exogenous or endogenous insult, due to epigenetic changes and not to permanent genetic rearrangements) (133–135), which challenges the historical assumption that the innate system is devoid of memory, was found to be closely interconnected with cellular metabolism (132). Given the important role of the innate system in the pathogenesis of NAFLD, as already proposed (75), TI could be an interesting subject of study in such a disease.

DAMPs and pathogen-associated molecular patterns (PAMPs) are released following NAFLD-associated damage and dysfunctions, they act as a signal of danger and can start an inflammatory process (136). PAMPs are exogenous danger signals made up of various microbially derived molecules, e.g., lipopolysaccharide (LPS), peptidoglycans, bacterial genetic material, etc., which can reach the liver due to the altered intestinal permeability associated with NAFLD (137). DAMPs correspond to endogenous molecules released by damaged cells, which can act as warning signals. DAMPs is a functional definition, and various molecules, with great diversity, are part of this family (83, 136), including HSPs, sp100 protein, HMGB1, DNA, RNA, etc. DAMPs and PAMPs are recognized by PRRs, which include TLRs, NOD-like receptors (NLRs), retinoic acid inducible gene I (RIG-I) -like receptors (RLRs), and others (132, 136). In this way, they activate cells of the innate immune cells, not purely immune cells, and DAMPs can also regulate adaptive immunity (138, 139). TLR4 (receptor for LPS) (55) and TLR9 (DNA) (140) were found implicated in NAFLD progression; TLR5 (flagellin) was hypothesized to have a protective role in liver disease induced by diet (141); TLR2 (cellular components of Gram-positive bacteria) has instead shown contradictory roles (142).

Some lipid species have also been shown that they can directly activate immune cells: saturated fatty acids (SFAs) were observed to induce COX-2 via TLR4 and NFκB in a macrophage-like cell line (143), as well as the activation of inflammatory responses mediated by macrophages and involving the liver was observed following exposure to peroxidized fatty acids (144) and free cholesterol (130, 145). As already mentioned, adipose tissue dysfunction in NAFLD was proposed as another factor inducing hepatic immune system activation, due to the imbalance in cytokine production (51, 137, 146).

In addition to the innate immunity, which was believed to play a prevalent role, adaptive cells are also greatly involved in NAFLD (147).

As DAMPs can initiate an inflammatory process without the participation of infective agents, they are actors of a sterile inflammation. More precisely, the inflammatory response which occurs in NAFLD is due to metabolic alterations, such as insulin resistance, excess of fat, and lipotoxicity, therefore it can be called “metabolic inflammation.” This process is characterized by a chronic low-grade immune activation, which does not resolve (148). This contrasts with an acute insult like microbial infection, in which the immune response is strong, limited in time, and has the purpose of eliminating the pathogen and making the person survive. Prolonged, unresolved, and low-grade inflammation gets no advantage to the host (149), and in NAFLD it causes the onset of scars responsible for liver cirrhosis. Differences in frequency and phenotype of several immune cells were described in NAFLD compared to healthy liver (150). Although the specific role of some of these in NAFLD is far from clear, it is likely that in addition to contributing to inflammation and disease progression, some elements play a protective role, e.g., NK cells through inhibitory cytokines and induction of apoptosis.

### Innate Immune System

The innate immunity is capable of very rapid, although not specific, responses and their subset are important players in the pathogenesis of NASH. As mentioned, non-strictly immune cells, such as hepatocytes, also are included in this field. Innate and innate-like cells predominate in the liver and constitute the first line of defense against danger signals.

#### Macrophages and Monocytes

The liver comprises the largest proportion (80–90%) of resident macrophages in the human body (151). The hepatic macrophages consist of different cell populations including the resident macrophages named Kupffer cells (KC) after their discoverer by Karl Wilhelm von Kupffer (152) and the infiltrating bone marrow derived monocytes (130, 153).

The KCs originate from the yolk sac and act as the dominant liver phagocyte. They localize inside the sinusoids directly in contact with blood circulation (154) and can migrate through the tissue along sinusoidal walls independently, and in different directions from those of neighboring Kupffer cells (155). The diverse origins of the macrophages reflect the high levels of phenotypical heterogeneity of this cell population (153, 156, 157). Recent studies, using single-cell RNA sequencing, revealed distinct hepatic macrophages with inflammatory and tolerogenic/non-inflammatory phenotypes (158, 159). The different macrophage populations are involved in both hepatic homeostasis and inflammation. KCs promote immune tolerance (160) and play a role in the early response to injury and infection (161), while the infiltrating macrophages are responsible for inflammation and fibrosis progression (153, 162).

Through the polarization process, the macrophages differentiate into subpopulations with specific biological functions. Simplifying, they can be divided into M1 macrophages with pro-inflammatory and antimicrobial activity and M2 with anti-inflammatory and reparative functions (153).

Both KCs and infiltrating monocytes play an essential role in various liver diseases. Several reviews have described their role in liver diseases, such as acute liver failure (163), liver fibrosis (164, 165), non-alcoholic fatty liver disease (130, 157, 159), viral hepatitis (166), and hepatocellular carcinoma (167, 168). Macrophages have been demonstrated to be implicated also in NAFLD development and severity (130, 161). In NAFLD subjects the infiltration of portal macrophages is observed at an early stage before the evidence of inflammation and their activation contributes to disease initiation and progression (169). Another study revealed an increase of activated KCs within the hepatic sinusoids in children with NASH (170). In addition, activated KCs modulate the severity of inflammation in NASH (171).

Alternatively, it was described also an anti-inflammatory role for hepatic macrophages; in fact, activated M2 macrophages can favor liver remodeling and tissue repair in NAFLD and initiate the apoptosis of inflammatory KCs (161). Moreover, NAFLD can increase the risk of development of HCC and tumor associated macrophages secrete inflammatory cytokines and growth factors involved in tumor development and progression. Toll-like receptor (TLR) 4 on macrophages has been shown to contribute to HCC proliferation (167, 172).

As macrophages play a central role in NAFLD, they might be a suitable target for therapies and a biomarker of diseases severity. In the liver, KCs produce the cytokine TNF-α in response to infections; elevated levels of TNF-α in patients without evidence of NAFLD have been demonstrated to be associated with a high risk of fatty liver development (173). Macrophages produce also other proinflammatory cytokines such as IL1 and IL18. Different studies have proven that IL-1α and IL-1β have a significant role in the progression of NAFLD (174, 175). Another cytokine potentially applicable in the diagnosis of NAFLD is IL-18, which is produced by macrophages and KCs. Circulating IL-18 levels correlate with metabolic syndrome (176), but, on the other hand, it has been also demonstrated that IL-18 production negatively regulates NASH progression *via* modulation of the gut microbiota (177).

Another cytokine secreted by KCs is TGF-β; the patients with elevated levels of isoform TGF-β3 show a higher risk of NAFLD development (178). Interestingly the soluble macrophage activation marker CD163 has been reported to correlate with liver injury and demonstrated good predictive ability for advanced fibrosis, which was further increased in combination with the NAFLD fibrosis score (179). However, this marker showed poor associations with liver histology in pediatric NAFLD subjects suggesting a possible different role for macrophages in the pathogenesis of adult and pediatric NAFLD (180). Another study demonstrated that the serum macrophage-derived deaminase ADA2 activity can predict NAFLD and liver fibrosis (181).

#### Dendritic Cells

Dendritic cells (DCs) have been described as interstitial and non-phagocytic cells. They localize periportally, around central veins and in the liver capsule (157). DCs function as antigen-presenting cells (APCs) recruiting other phagocytic cells to the injury site. DCs play an important role to initiate the immune response by capturing, processing, and presenting the antigens to T cells (182). During homeostasis, DCs display a predominant tolerogenic and immature phenotype. While, in the context of inflammatory state, they maturate and enhance the production of proinflammatory cytokines. Mature DCs activate natural killer T cells and promote T-cell proliferation (183). In NASH mice models hepatic DCs exhibit increment of the production of pro-inflammatory cytokines and chemokines (171). Liver DCs are also implicated in adipogenesis, lipid metabolism and synthesis, and hepatic accumulation (184). Human hepatic DCs are composed of two distinct populations that contain different concentrations of lipid, which regulates immunogenic vs. tolerogenic responses. The increased concentration of toxic lipid plays an important role in the pathogenesis of acute and chronic liver diseases (160, 185).

#### Neutrophils

Neutrophils are the most abundant group of white blood cells circulating in healthy adults and a key component of the innate response. These cells, which have a limited life span (1–2 days), act by phagocytosis, the release of substances (defensins) contained in their granules including neutrophil elastase (NE), myeloperoxidase (MPO), and lysozyme, the production of reactive oxygen species (ROS), and through the NETs (neutrophil extracellular traps) (186). In addition to microbial invasion, metabolic insults can also induce the recruitment and activation of neutrophils (187). In fact, they are part of the inflammatory infiltrate which characterizes the histology of NAFLD (169), and the extent of the infiltration was found to correlate with the severity of the disease (187). They migrate from the blood circulation to the focus of the inflammation, driven by chemokines and chemotactic agents, which are released creating a gradient within the hepatic compartment (188). Neutrophils are among the first cells to invade the liver in NAFLD, and in this site can attract other immune cells (187, 189). The invasion begins soon after damage, following the release of DAMPs by the damaged hepatocytes (190); furthermore, danger signals derived from the gut also contribute to the recruitment and activation of neutrophils in NAFLD (191). In NASH it was documented a hepatocyte upregulation of the main chemokines that attract neutrophils (186). Neutrophil-to-lymphocyte ratio (NLR) has been observed to correlate with advanced inflammation and fibrosis in NAFLD patients (192). Moreover, NAFLD patients showed an increase in MPO (193), NETs (194), NE, and PR3 (195) circulating levels. The hepatic concentrations of the latter were associated with advanced stages of the disease (195). MPO showed that it could activate HSCs promoting liver fibrogenesis; its pro-fibrogenic role was also linked to the induction of polarization to M2-macrophages (186, 191). Furthermore, NE showed to be a regulator of insulin signaling, and its deletion improved insulin sensitivity in a mouse model of obesity (196).

#### Natural Killer Cells

Natural killer cells (NK cells) belong to the innate immune system and act through the production of granules containing perforin and granzymes (197), but they can also play an important role in shaping the adaptive immune response (198). In the liver, they are found within the hepatic sinusoids. These cells can be distinguished into CD56^dim^ NK cells (which represent the most abundant group in peripheral blood) and CD56^bright^ (197). NKG2D is an activating receptor expressed by NK cells, but also by others of the immune system such as T lymphocytes, and is involved in the identification and elimination of damaged cells (199), so acting as a receptor for danger signals. *In vitro* and *in vivo* models showed that NK cells can kill human and mouse HSCs by mechanisms dependent on RAE1, NKG2D, TRAIL, NKp46/NCR1, and p38/PI3K (200–202). In a study of NAFLD patients, NASH ones were found to have higher hepatic levels of NK cells and NKG2D mRNA (203). Furthermore, the NK cells showed different levels of activation based on the levels of fibrosis. CD56^dim^ NK cells circulating levels were found to be high in advanced fibrosis (F3/F4) than in healthy controls, differently from patients with early stages of the disease; moreover, they were found in an inactive state in patients with NAFLD and advanced fibrosis (204). The increased number observed in advanced disease was hypothesized to be a compensatory event to NK cells impairment. For these reasons, NK cells have been linked to a protective role in liver fibrosis.

### Innate-Like, “Unconventional,” T Lymphocytes

Mucosal-Associated Invariant T (MAIT) cells are currently defined as MR1-Ag restricted cells which have a TCR including Vα7.2 segment paired with Jα33, Jα12, or Jα20; these α-chains associate with a limited repertoire of β-chains. The most studied antigen which MAIT cells recognize by their TCR is a metabolite of riboflavin biosynthesis (205). In healthy people, circulating MAIT cells are 1–10% of total T cells, whereas in the liver they reach up to 45% of intrahepatic T lymphocytes. They are generally CD3+, DN or CD8+, Vα7.2+, CD161+, IL-18Rα+, CD26+, PLZF+ (205). It was observed that in patients with NAFLD related cirrhosis circulating levels of MAIT cells were reduced; in the same study MAIT cells were found to cause proliferation of human hepatic myofibroblasts (HMFs) and release of proinflammatory cytokines by HMFs and macrophages; moreover, CCl4-exposed MAIT cell-deficient mice resulted protected from fibrosis whereas CCl4-exposed MAIT cell-enriched mice showed an increase in fibrosis (compared with WT ones) (206). Another study showed that circulating MAIT cells were reduced and functionally impaired (decreased production of IFN-γ and TNF-α), in NAFLD patients; MAIT cells were increased in the liver of NAFLD patients, and their number was found to positively correlate with the NAS values (NAFLD activity score); *in vitro*, activated MAIT cells induced macrophages differentiation toward M2 phenotype, and MAIT cells-deficient MCD-fed mice showed enhanced liver steatosis and inflammation than WT mice, thus suggesting a protective role for these cells in disease progression (207). Given the conflicting results and the limited availability of studies, the role of MAIT cells in NAFLD appears still unclear.

Natural killer T (NKT) cells are CD1d restricted lymphocytes, which recognize lipid antigens. This definition is due to their expression of both the classic T lymphocyte (CD3) and natural killer cell markers (e.g., CD56) (148). These cells can be divided into two subtypes: invariant NKT (iNKT), or NKT type 1, which possess a semi-invariant TCR-α chain (which in humans includes the Vα24/Jα18 region), and type 2, non-invariant NKT (type 2), with a more variable TCR. They produce cytokines associating with T helper 1 and T helper 2 cells, and also utilize Fas and TNF-a to induce apoptosis, guiding the immune system into tolerance or inflammation (208). Regarding the role of NKTs in NAFLD, contradictory data emerged on their effects on hepatic steatosis, inflammation, and fibrosis. In fact, it was observed that in wild-type mice fed with MCD diets, NKT cells had a profibrogenic role by production of osteopontin (OPN) and hedgehog (Hh) ligands, and by activation of HSCs (209). In another study, reduced steatosis, fibrosis, HSCs activation, and hepatic infiltration of inflammatory cells were observed in iNKT cell–deficient mice on CDAA diet (210). However, improvement in NASH associated with an increase in the intrahepatic population of NKT in leptin-deficient ob/ob mice model (211), increase in liver fat in CD1d −/− (lacking NKT cells) mice following HFD (212), and of liver inflammation and fibrosis in iNKT-lacking, HFD-fed mice (193), were also observed.

γδ-T cells express a TCR formed by γ and δ chains (instead of α and β) and are another T cell population, which can be found in the liver. This group represents 15–25% of all intrahepatic T cells (213) and was predominantly found in portal infiltrates and areas of bile duct proliferation or fibrogenesis (214). These cells recognize non-peptide bacterial antigens, and other ligands, and are IL17A producers (215). γδ-T cells were observed to be increased in the liver of HFD-induced obesity and NAFLD mice; reduced liver damage and steatohepatitis were observed in γδ T cell-deficient mice. Moreover, the gut microbiota showed to support disease progression by γδ-T IL17+ cells (216). In another study on MCD-fed mice, it was observed that γδ-T depletion protects against steatohepatitis, thus demonstrating their pathogenetic role in NAFLD; in this work, however, the progression of the disease appeared IL-17 independent (217). Further studies are needed to clarify the effect of these cells in the progression of NAFLD.

### Adaptive Immune System

Adaptive immune cells are recruited by events initiated by innate immunity, but they trigger a more effective, specific response. Although innate immunity has been considered a key player in NAFLD, recent evidence also sheds light on the adaptive system in this condition. After all, NASH is characterized by an intense lymphocytic infiltrate (148), and aggregates of both T and B lymphocytes can be found in NAFLD (218, 219).

#### CD4+ Helper T Lymphocytes

CD4+ T lymphocytes are further divided into subpopulations based on their functions and cytokines production (220). Among these, there are Th1 cells (proinflammatory cells with a critical role in defense against intracellular pathogens, producing IFN-γ, IL-2, TNFα), Th2 (involved in allergic diseases and response against parasites, producing IL-4, IL-5, IL-13, IL10), Th17 (proinflammatory cells with a defensive role against extracellular bacteria, but also fungi, producing IL17A, IL17F, IL21, IL22, IL23), Th22 (antibacterial functions, producing IL-22), Treg (key elements in the maintenance of self-tolerance, suppressing T-cell activation and releasing IL-10, TGF-β, IL-4) (220). Liver recruitment of CD4+ T lymphocytes was observed in patients and mice models of NASH (221, 222). It was observed that methionine and choline-deficient high-fat (MCDHF) fed, IFN-γ-deficient mice showed less steatosis, inflammation, and fibrosis than WT counterparts. In the same study, it was also observed, *in vitro*, that IFN-γ induced TNF-α production by macrophages in a dose-dependent manner (223). Other studies also suggested a role of Th1 in NAFLD, showing an increase of these cells, Th1 proinflammatory cytokines, or genes toward a Th1 phenotype polarization in patients with NASH (218, 224, 225). In a study of 112 patients with NAFLD (of whom 51 had biopsy-proven NAFL and 30 biopsy-proven NASH) a higher frequency of IFN-γ+ and/or IL-4+ cells was observed in peripheral blood of patients with NAFL and NASH than healthy controls, and a marked increase in intrahepatic IL-17, IL-4, and IFN-γ-producing T cells in NAFLD patients, compared to peripheral blood. In addition, an increase in activation of CD4+ T lymphocytes was documented both in peripheral blood and liver (based on the expression of HLA-DR) (226); Th17 was found to be more abundant in the liver of patients with NASH than in those with NAFL and in circulating blood of NASH patients Th17/Treg ratio was found to be higher than that of NAFL ones. These difference, as well as the histology improved, was found attenuated 1 year after bariatric surgery. Therefore, the authors hypothesized that the balance between Th17 and Treg plays a key role in the pathogenesis of NASH (226). Temporal changes in the frequency of T CD4 lymphocyte populations during NAFLD progression have also been observed: in a study on MCD-fed mice, it was observed an increase in Th17 cells in the first phases of the disease, and in the NASH-fibrosis transition, while Th22 increased between the two Th17 expansions. In the same study, an *in vitro* model of hepatocyte lipotoxicity documented that IL-17 exacerbated, while IL-22 prevented hepatocyte lipotoxicity (221). The pathogenetic role of IL-17 in progression from NAFL to NASH has also been documented in other studies (227), while the role of IL-22 in chronic liver disease is not so clear (228). IL-17 has been shown to be able to stimulate Kupffer cells to produce inflammatory and fibrogenic cytokines (including TGF-β) and to directly stimulate HSCs by promoting their activation and the production of type 1 collagen by STAT3 (229). For these reasons, as stated in a recent review, Th1 and Th17 lymphocytes are generally attributed a pathogenetic role in the progression of NAFLD (218).

#### CD8+ Cytotoxic T Lymphocytes

These effector cells act by releasing cytokines, cytolytic substances such as perforin and granzymes, and cell-cell contact. Cytotoxic T lymphocytes increase in the liver of people with NAFLD, where they are more activated. Their depletion was observed to be associated with a reduction in steatosis, inflammation, fibrosis, and insulin resistance (148, 220, 230). Furthermore, CD8 + T lymphocytes (as well as NKTs) were found to promote the transition from NASH to HCC (231). Their role in the progression of NAFLD, however, needs to be better investigated with further studies.

#### B Lymphocytes

B lymphocytes are responsible for various immunological functions, including production of antibodies, antigen presentation, cytokines secretion, and regulation of immune responses. However, their biological function in the liver is still not fully elucidated. Only a small number of B cells are residing in the healthy liver and, maybe since hepatic B cells comprise only ~5% of intrahepatic lymphocytes, there are experimental difficulties in isolating and analyzing specifically these cells (232). This lymphocytes population has been shown to infiltrate the liver parenchyma of NASH patients. These cells may contribute to the progression of the disease through the production of inflammatory mediators and antigen presentation (218); they showed to exert a profibrogenic role through the release of inflammatory cytokines stimulating HSCs (233). In mouse models of NAFLD, it was observed that B lymphocytes were activated early in the course of the disease and resulted important for recruitment and activation of T lymphocytes (219). Circulating levels of the cytokine BAFF were found to be higher in patients with NASH than in those with simple steatosis, and the higher levels of this cytokine correlated with hepatocyte ballooning and advanced fibrosis (234). In a study in which biopsy-proven NAFL and NASH patients had serum immunoglobulin measurements, it was also observed that IgA levels were elevated more frequently in NASH patients compared to those with simple steatosis (235).

## Therapeutic Approaches Acting on the Immune System to Counter the Progression of NAFLD

Several drugs have been studied to reduce liver inflammation and fibrogenesis in NAFLD, resolution of steatohepatitis and improvement in liver fibrosis representing two key endpoints of current trials (236). Moreover, it is also being studied the effect of the combination of molecules acting on different targets. However, despite the advances in knowledge of the fibrogenic process leading to cirrhosis, to date there are no approved and specific pharmacotherapy to resolve NASH, and targeting the predisposing factors (by lifestyle modifications and weight loss) is considered the best therapeutic option (237). The regression of fibrosis is already obtainable in some conditions, such as in chronic viral hepatitis after antiviral therapy, or for obese NAFLD patients, following bariatric surgery (65, 238). Given the key role of the immune system in the progression of NAFLD, therapeutic approaches aimed at counteracting its harmful role in pathogenesis have also been tested (239). Although many extensively examined or new molecules under study for NAFLD not acting directly on the immune system cells, for example having a primary antioxidant effect (e.g., vitamin E) (240), acting on bile acid metabolism [e.g., OCA, which is the most advanced molecule in the race for drug approval to treat NASH (236)] or on glucose or lipid metabolism (e.g., Elafibranor), or having other primary targets, spill over their action to the immune system (86), below they will be summarized only approaches directly engaging the immune substrate of NAFLD (a list is provided in Table 1).

**Table 1:** Summary of the drugs recently studied for NAFLD therapy which have a mechanism of action that involves immune system modulation.

**Drug name (study reference)**	**Drug type**	**Mechanism of action**	**Expected effect**	**Administration route**	**Experimental stage reached**	**Efficacy**	**Future perspectives**
Cenicriviroc (245)	C-C chemokine receptor type 2 and 5 antagonist	Reduction of migration of monocytes/macrophages, reduction of HSCs activation	Antinflammatory, antifibrotic	Daily oral route	Phase-3 double blind RCT	Stopped for lack of efficacy	Not approved in monotherapy, association with Tropifexor ongoing
Belapectin (GR-MD-02) (252)	Galectin inhibitor	Reduction of galectin secretion with reduction of neutrophils adhesion, opsonization, macrophage chemoattraction, myofibroblast activation	Antinflammatory, antifibrotic, portal hypertension reduction	Intravenously	Phase-2b double blind RCT	Only efficacious in reducing HVPG in pts without esophageal varices at baseline	Phase 2b/3 trial on the efficacy on preventing varices in NASH cirrhosis pts without varices ongoing
Protexin capsules (256)	Synbiotic supplement (prebiotic and probiotic)	Attenuation of inflammatory responses	Antinflammatory, antifibrotic	Daily oral route	Double blind RCT	Improved liver biochemistry, reduced transient elastography score	Available for clinical use, effects of longer treatment durations remain to be determined
Symbiter (257)	Multi-probiotic	Reduction of the inflammatory response and hepatic triglycerides content	Antisteatosic, antinflammatory, antifibrotic	Daily oral route	Double blind RCT	Reduced liver fat, AST, GGT, TNF-α, and IL-6 in NAFLD patients	Available for clinical use, long-term studies required
JKB-121 (264)	TLR-4 antagonist	Reduction of TLR-4 mediated liver inflammation and fibrosis	Antinflammatory, antifibrotic	Twice daily	Phase 2b RCT	JKB-121 did not perform better than placebo in improving liver fat content and/or serum ALT in NASH patients	Further studies on the inhibition of TLR-4 are needed
GPR84 Antagonist (266)	GPR84 antagonist	Inhibition of inflammatory responses GPR84 mediated	Antinflammatory, antifibrotic	Orally administered	Preclinical (mouse) NAFLD model	Reduced macrophages and neutrophil infiltration, ameliorated steatohepatitis	Further studies needed
BI 1467335 (271)	VAP-1 inhibitor	Reduction of hepatic accumulation of inflammatory cells	Antinflammatory, antifibrotic	Oral tablets	Phase 2 RCT	Improved NASH biomarkers	Development discontinued (risk of drug interactions)
Sandy-2 (219)	B-cell Activating Factor (BAFF) -neutralizing monoclonal antibody	Prevention of B cells maturation	Antinflammatory, antifibrotic	I.p. injection	Preclinical (mouse) NASH model	Prevented hepatic B cell maturation, reduced Th-1 lymphocytes activation, ameliorated steatohepatitis	Further studies needed
OKT3 Mab (274)	Anti-CD3 monoclonal antibody	Immunomodulatory effect, induction of regulatory T cells (Tregs)	Antinflammatory, antifibrotic	Oral once daily	Phase 2a RCT	Improved liver, metabolic, and immunologic parameters	Further trials are needed

Cenicriviroc (CVC) is a C-C chemokine receptor type 2 and 5 (CCR2 and CCR5) antagonist, expressed mainly on monocytes the former, and on various immune system cells (including lymphocytes) and HSCs the latter. Following the recognition of their ligands, these receptors participate in the recruitment and activation of various immune cells, which were linked to amplification and perpetuation of the inflammatory response in NAFLD (241). Therefore, the rationale for the use of CVC was a reduced migration and hepatic infiltration of monocytes/macrophages (due to the blockade of CCR2), and a reduced migration and activation of HSCs (due to the parallel inhibition of CCR5). Preclinical studies have shown its effectiveness in reducing liver fibrosis (241). In a study (CENTAUR trial) involving 289 subjects with NASH and hepatic fibrosis in which 145 received CVC and 144 placebo, it was observed that it was safe and well-tolerated, but the primary outcome of improvement in NAS by ≥2 points without worsening of fibrosis after 1 year, was not met. However, this drug improved liver fibrosis in a significantly higher percentage of cases than placebo (20 vs. 10%) (242). After 2 years of treatment, most people who achieved improvement in fibrosis maintained this result (243). It was being tested in a randomized, double-blind, placebo-controlled phase 3 trial (AURORA) to evaluate its efficacy in the treatment of liver fibrosis in adults with NASH (244), but this study was stopped early due to lack of efficacy (245).

Galectin inhibitors are a class of compounds that interfere with galectins. The latter are carbohydrate-binding proteins that are located inside the cells, in the cytoplasm, in states of quiescence, but can be externalized. In fact, in case of tissue damage, the cytosolic galectins are actively secreted by the cells, and act as DAMP. The main galectin produced during damage is Galectin-3 (Gal-3), which is primarily produced by macrophages (246). It is involved in several inflammatory processes, including the adhesion of neutrophils, opsonization, and macrophages chemoattraction (247, 248). Moreover, Gal-3 was found to lead to myofibroblast activation (249), and was linked to the fibrogenesis process in different liver diseases (246). Galectin-3 inhibitors resulted effective in preclinical studies of NASH and liver fibrosis (250). Among the galectin inhibitors, there is belapectin (GR-MD-02), a natural plant derived molecule that binds to Gal-3 (but also to galectin-1). In a phase 1 study, GR-MD-02 was shown to be safe and well-tolerated in patients with NASH or advanced fibrosis proven by biopsy (251). Therefore, its efficacy was studied in a randomized placebo-controlled trial in patients with liver cirrhosis and portal hypertension; 162 participants were randomized to receive belapectin, 2 or 8 mg/kg, or placebo, but neither dose was found to reach the primary endpoint (HVPG reduction), nor improve liver fibrosis, or reduce the incidence of complications of cirrhosis. However, this drug showed to be associated with an improvement in hepatocyte ballooning. It was also observed that belapectin could have a favorable effect on HVPG and the development of varices in a specific group of patients (NASH-cirrhosis without varices at baseline) (246). A study to evaluate the safety and efficacy of belapectin vs. placebo for the prevention of esophageal varices in patients affected by NASH cirrhosis with signs of portal hypertension but without esophageal varices (NAVIGATE) is currently ongoing (252).

Hepatic macrophages are an interesting target for novel therapeutic approaches for liver diseases. However, there are some important challenges to be faced, like the quite opposing functions of macrophage subsets depending on the experimental condition observed in the animal models, the not complete comparability between animal and human diseases, and the complex human macrophages heterogeneity. However, the increasing understanding about macrophages allowed the identification of several pathways that regulate their recruitment, differentiation/polarization and activation, offering promising starting points for novel therapeutic intervention. Different approaches include inhibition of KCs activation, dampening of monocyte recruitment into the liver, and modulation of macrophage polarization/differentiation. KCs activation can be influenced by several approaches. Using antibiotics, it is possible to reduce the bacterial infection and the consequent TLR4-dependent macrophage activation, ameliorating steatohepatitis, fibrosis, and hepatocarcinogenesis in mice models (253, 254). Antibiotics act influencing the gut barrier and microbiota. Also the probiotics could potentially alleviate pathogenic Kupffer cell activation in the liver (255). Probiotics have several beneficial properties, including interaction with the enterohepatic axis. It has been shown that the use of preparations containing different strains of bacteria and a probiotic in patients with NAFLD is associated with a significant reduction of hs-CRP, TNF-α, and TNFκ-B p65 (256). Beneficial effects of other multiprobiotic compounds have been observed in patients with NAFLD (257). Inflammatory monocytes recruitment to the liver is driven by chemokines. Therefore, different pharmacological strategies have been generated to interfere with chemokine signaling, including monoclonal antibodies, receptor antagonists, inhibition of chemokines (258); an example of this type of pharmacological approach is the aforementioned cenicriviroc. KCs have a high scavenging capacity, which can be used for drug delivery. In fact, dexamethasone has been demonstrated to reduce fibrosis in mice models through a macrophage-targeted delivery (259, 260). A fascinating alternative to treat liver disease in a murine model is the infusion of KCs expanded *in vitro* to ameliorate liver fibrosis (261). Moreover, macrophages can be isolated from apheresis derived CD14 monocytes of cirrhotic patients and differentiated into macrophages with a pro-resolution phenotype (262, 263).

JKB-121 is an antagonist of TLR-4, which was linked to liver inflammation and fibrosis. Encouraging results derived from preclinical studies on the antagonism of TLR4, but, in a trial on patients with biopsy-proven NASH, grade 1-3 fibrosis, and hypertransaminasemia JKB-121 did not reach the endpoint of reducing the liver fat content by MRI-PDFF and/or serum ALT after 24 weeks (264).

GRI0621, a natural killer T (NKT) cells antagonist, has been investigated in a study on patients with chronic liver disease including NASH to test its effects, but the study was discontinued for administrative decision (265).

G protein-coupled receptor 84 (GPR84) is a surface receptor for medium-chain fatty acids (MCFA). This receptor is expressed by several cells of the innate immune system and showed proinflammatory functions (266). In GPR84-deficient mice, LCFA diet did not cause an increase in liver mass as was observed in WT counterparts (267). In a recent study, it was observed that GPR84 expression was increased in the liver of mice and humans with NAFLD and was associated with inflammation and fibrosis; GPR84 antagonists were found to reduce chemotaxis of monocytes and neutrophils. Moreover, these molecules showed to reduce macrophages accumulation and to improve inflammation and fibrosis in mouse models of NASH. The therapeutic effects in ameliorating steatohepatitis and fibrosis of GPR84 antagonists were linked to the inhibition of the migration of myeloid cells, and not to effects on HSCs, which were not found to express GPR84 (266). Further studies are needed to validate the effectiveness of targeting this system.

Vascular adhesion protein-1 (VAP-1) is a glycoprotein, which has amine oxidase activity and is involved in endothelial adhesion and transmigration processes of leukocytes (268). There is also a soluble form of VAP-1 (sVAP-1), whose levels were found to be elevated in patients with cardiovascular, metabolic (e.g., diabetes and obesity) and hepatic diseases (269). In the liver, it was found involved in the adhesion and transendothelial migration (through the sinusoids) of lymphocytes. It has been observed that sVAP-1 is increased in NAFLD patients and that VAP-1 hepatic expression is increased in patients affected by steatohepatitis compared to those with simple steatosis (269). Moreover, in mouse models of liver damage, inhibition of VAP-1 (by VAP-1–deficient mice or VAP-1 neutralizing antibodies) reduced hepatic migration of inflammatory cells (T cells, NKT cells, and myeloid cells) and attenuated fibrosis (269). Results of another study suggested that VAP-1 may contribute to the progression of NAFLD (270). Hence it has been proposed as a target to limit the progression of NAFLD. A phase II trial was started to document the effects of inhibiting VAP-1 (BI 1467335) in patients with NASH (271); however, the research company announced that it has stopped developing this molecule in NASH due to the risk of drug interactions^1^.

The role of B lymphocytes in the progression of NAFLD has been documented (218). Furthermore, the cytokine B-cell Activating Factor (BAFF), necessary for survival and maturation of B lymphocytes, has also been studied in patients with NAFLD. Circulating BAFF levels were found to be higher in patients with steatohepatitis than in those with NAFL (234). BAFF neutralization through BAFF-neutralizing monoclonal antibody Sandy-2 was shown to improve steatosis, inflammation, and fibrosis in transgenic (NASH model) mice overexpressing a soluble form of a BAFF/APRIL receptor (TACI-Ig) (219).

CD3 molecule is associated with the TCR receptor, and this complex is found on the surface of T lymphocytes. Unlike TCR, CD3 is not variable. Muromonab (OKT3) has been the first approved monoclonal antibody and was used to treat organ transplant rejection, but its application is limited by high toxicity. Hence, humanized anti-CD3 antibodies were developed to improve tolerability (272). In a preclinical study on ob/ob mice, anti-CD3 mAb showed to reduce liver fat, adipose tissue inflammation, and blood glucose (273). OKT3 was tested at different dosages (0.2, 1.0, 5.0 mg/day) in a phase II trial in patients with biopsy-proven NASH to determine its effects. This drug, administered for 30 days, was well-tolerated and led to the induction of regulatory T lymphocytes (274).

## Conclusions

Inflammation and fibrogenesis in NAFLD are multifactorial processes involving a multitude of interrelated mechanisms, and in which the immune system plays a key role. Although challenges about its pathogenesis still exist, the knowledge on NAFLD is increasing, leading, also for this reason, to the recent proposal of rename. Therapies aimed at directly fighting the immune substrate of NAFLD are already being studied. In any case, the precise characterization of some elements of the immune system has only occurred in recent years, and the specific role of many subsets in the pathogenesis of NAFLD, as well as that of many other human diseases, is still far from clear. Furthermore, the relationship between cellular metabolism and immune cell functions, termed “immunometabolism,” is a candidate for future studies in the field of NAFLD. This knowledge could allow scientists to further elucidate the pathophysiology of this complex disease and to hypothesize new therapeutic approaches.

## Author Contributions

PT and BMM wrote the paper. All authors participated in revising the manuscript.

## Conflict of Interest

The authors declare that the research was conducted in the absence of any commercial or financial relationships that could be construed as a potential conflict of interest.

## Publisher's Note

All claims expressed in this article are solely those of the authors and do not necessarily represent those of their affiliated organizations, or those of the publisher, the editors and the reviewers. Any product that may be evaluated in this article, or claim that may be made by its manufacturer, is not guaranteed or endorsed by the publisher.

## Footnotes

^1^Available online at: https://www.boehringer-ingelheim.us/press-release/boehringer-ingelheim-discontinues-development-bi-1467335-nash.
